# 63 General practitioners’ experience, attitudes and needs on providing advice on physical activity to people with chronic ischemic heart disease - A qualitative study (OptiCor study)

**DOI:** 10.1093/eurpub/ckae114.071

**Published:** 2024-09-26

**Authors:** Alicia Prinz, Verena Leve, Elisabeth Gummersbach, Stefan Wilm, Sabrina Hoppe, Franziska Vogl, Ursula Kirchhof, Anja Neuhaus, Sabrina Kastaun

**Affiliations:** Institute of General Practice (ifam), Patient-Physician-Communication Unit, Centre for Health and Society (chs), Medical Faculty and University Hospital of the Heinrich-Heine-University Düsseldorf, Düsseldorf, Germany; Institute of General Practice (ifam), Centre for Health and Society (chs), Medical Faculty and University Hospital of the Heinrich-Heine-University Düsseldorf, Düsseldorf, Germany; Institute of General Practice (ifam), Centre for Health and Society (chs), Medical Faculty and University Hospital of the Heinrich-Heine-University Düsseldorf, Düsseldorf, Germany; Institute of General Practice (ifam), Centre for Health and Society (chs), Medical Faculty and University Hospital of the Heinrich-Heine-University Düsseldorf, Düsseldorf, Germany; Institute of General Practice (ifam), Patient-Physician-Communication Unit, Centre for Health and Society (chs), Medical Faculty and University Hospital of the Heinrich-Heine-University Düsseldorf, Düsseldorf, Germany; Institute of General Practice (ifam), Patient-Physician-Communication Unit, Centre for Health and Society (chs), Medical Faculty and University Hospital of the Heinrich-Heine-University Düsseldorf, Düsseldorf, Germany; Patient representative of the OptiCor project and representative of the German Heart Foundation e.V., Düsseldorf, Germany; Patient representative of the OptiCor project and lead of the heart discussion group Düsseldorf, Düsseldorf, Germany; Institute of General Practice (ifam), Patient-Physician-Communication Unit, Centre for Health and Society (chs), Medical Faculty and University Hospital of the Heinrich-Heine-University Düsseldorf, Düsseldorf, Germany

## Abstract

**Purpose:**

The German treatment guideline “chronic ischemic/coronary heart disease (IHD)” recommends that general practitioners (GPs) deliver advice on physical activity (PA) to IHD patients. However, the provision of PA advice is inadequately implemented in general practice in Germany. One reason is the lack of medical training in providing PA advice effectively and efficiently. International guidelines recommend such training for GPs. This study aims to explore needs, experiences, and attitudes including barriers and facilitators of GPs towards the routine delivery of PA advice to IHD patients, in order to adequately inform a customised development of such a training.

**Methods:**

Over a period of four months (March – June 2023), 12 face-to-face problem centred interviews (n = 12; 5/12 females, age range: 35-73 years) and six focus group discussions (n = 37; 13/37 females, age range: 36-70 years) with GPs were conducted. Interview and discussion guides were developed and pilot tested by the multi-professional study team. Data were collected in an iterative process. Purposive sampling and techniques of maximal structural variation were applied (e.g., with regard to professional experience, interest in PA). Audio-recorded data were transcribed verbatim and analysed using a content structuring procedure (deductive and inductive approach). GPs were involved throughout the entire research process, e.g., in multi-professional analysis groups.

**Results:**

Whereas GPs are mostly aware of the health benefits of PA for IHD patients, PA advice is not routinely provided. Conversations on PA tend to be unstructured and advice is often addressed more generally than customised to the patients’ needs and preferences. Priority is given to other lifestyle issues, such as smoking. The effectiveness of PA advice is perceived as less effective but time consuming. GPs mention frustration in this context. They express a need for communication strategies (structure and tools) to motivate patients to PA that can be integrated into everyday GP care.

**Conclusion:**

The results provide relevant insights into the everyday practice of GPs, information on GPs’ needs and requirements and can thus contribute to a target group-specific development of a GP training on PA advice for patients with IHD in the OptiCor study.

**Funding:**

German Ministry of Education and Research (01GY2103).

